# Comparison of Social Media, Syndromic Surveillance, and Microbiologic Acute Respiratory Infection Data: Observational Study

**DOI:** 10.2196/14986

**Published:** 2020-04-24

**Authors:** Ashlynn R Daughton, Rumi Chunara, Michael J Paul

**Affiliations:** 1 Analytics, Intelligence and Technology Los Alamos National Laboratory Los Alamos, NM United States; 2 Biostatistics School of Global Public Health New York University New York, NY United States; 3 Computer Science and Engineering Tandon School of Engineering New York University Brooklyn, NY United States; 4 Information Science Department University of Colorado Boulder Boulder, CO United States

**Keywords:** social media, infodemiology, influenza, human, selection bias, bias, logistic models

## Abstract

**Background:**

Internet data can be used to improve infectious disease models. However, the representativeness and individual-level validity of internet-derived measures are largely unexplored as this requires ground truth data for study.

**Objective:**

This study sought to identify relationships between Web-based behaviors and/or conversation topics and health status using a ground truth, survey-based dataset.

**Methods:**

This study leveraged a unique dataset of self-reported surveys, microbiological laboratory tests, and social media data from the same individuals toward understanding the validity of individual-level constructs pertaining to influenza-like illness in social media data. Logistic regression models were used to identify illness in Twitter posts using user posting behaviors and topic model features extracted from users’ tweets.

**Results:**

Of 396 original study participants, only 81 met the inclusion criteria for this study. Of these participants’ tweets, we identified only two instances that were related to health and occurred within 2 weeks (before or after) of a survey indicating symptoms. It was not possible to predict when participants reported symptoms using features derived from topic models (area under the curve [AUC]=0.51; *P*=.38), though it was possible using behavior features, albeit with a very small effect size (AUC=0.53; *P*≤.001). Individual symptoms were also generally not predictable either. The study sample and a random sample from Twitter are predictably different on held-out data (AUC=0.67; *P*≤.001), meaning that the content posted by people who participated in this study was predictably different from that posted by random Twitter users. Individuals in the random sample and the GoViral sample used Twitter with similar frequencies (similar @ mentions, number of tweets, and number of retweets; AUC=0.50; *P*=.19).

**Conclusions:**

To our knowledge, this is the first instance of an attempt to use a ground truth dataset to validate infectious disease observations in social media data. The lack of signal, the lack of predictability among behaviors or topics, and the demonstrated volunteer bias in the study population are important findings for the large and growing body of disease surveillance using internet-sourced data.

## Introduction

### Background

Internet data have been used in several contexts to improve infectious disease surveillance and prediction for many diseases, including influenza [1], cholera [2], dengue [3], and malaria [4]. They have been shown, in some instances, to be predictive of the incidence of infectious diseases (eg, seasonal influenza [1,5-8]), but there are also cases where the data hold little predictive value [8,9]. In general, disease prediction based on internet-sourced data utilize explicit mentions of symptoms or references to illness. Some have identified symptom reports from social media data using machine learning classifiers [7,10]. Others use search queries related to a disease of interest [1,11,12]. These counts are then used to infer population-level statistics such as from the US Centers for Disease Control and Prevention. A major advantage is that internet data can be obtained in real time, whereas traditional public health data can take weeks or even months to compile [13]. Research has also found that the combination of internet data with traditional sources can improve forecasts [6,14].

Only recently has data representativeness been explored. Some works have explored the degree to which social media users are representative of the broader population [15,16] or methods to account for biases [17]. However, little is known about the validity of health information such users share on the internet and conversely how information is shared when users are actually sick. In other words, although social media disease detection systems have been validated against official reports at the population level [10,18], the relationship between population-level models and individual-level information is not well understood—for example, how often do social media users post that they are sick, and in what ways?

Answering these questions requires data about individuals. Although infectious disease research with internet data has largely not had access to ground truth datasets, there is substantial prior work in related domains. Researchers have used crowdsourcing methods to create ground truth datasets of individuals with clinical depression [19], compared electronic medical records with topics of Facebook posts [20], and used self-disclosures of attention deficit hyperactivity disorder on the internet as ground truth datasets [21].

This study sought to advance our understanding of the relationship between people’s actual health statuses and their social media activity. We used influenza-like illness and similar syndromes, such as the common cold, as a case study. We leveraged individual-level health data, including weekly symptom self-reports and viral diagnostic data collected through the GoViral platform, an internet-based influenza-like illness surveillance system, in which participants returned a weekly symptom survey when sick. Data from these reports were used alongside Twitter messages posted by the individuals (each individual considered here also shared their public Twitter profile information). Our experiments examined how often and in what ways individuals tweeted about their health in relation to the health status described by their survey responses. We measured whether survey-derived health statuses can be predicted with social media–derived variables about individuals and if the study participants differed predictably from Twitter users.

### Objectives

Specifically, we answered three research questions (RQs).

- RQ1: How often and in what ways do people share their illnesses on Twitter when they are ill?
- RQ2: How predictable is someone’s illness status from their tweets?
- RQ3: How are results from individuals in the GoViral study potentially representative (or not representative) of Twitter users more generally?

RQ1 and RQ2 sought to improve our understanding of the relationship between an individual’s health status and social media activity, whereas RQ3 seeks to understand how representative were the data we used.

## Methods

### Data Collection

The GoViral platform was developed to generate self-reported symptoms and biospecimens from a cohort of lay volunteers. Although the GoViral platform had been in operation since November 2013, the operationalizing of Twitter handle collection commenced in August 2016. This study includes data from participants recruited between August 2016 and November 29, 2017.

Recruitment, eligibility, and enrollment procedures remained consistent with the existing platform. Enrollment was driven largely by recruitment in person at relevant community outposts and events. Paid online advertisements and social media were also used as a means of recruiting volunteers to the study. The study size was limited by the ability to recruit and engage participants. To register, volunteers signed an electronic consent form and reported their email address, name, mailing address, gender, and age. Volunteers were sent a kit that included collection materials and customized instructions to keep at home. Users were instructed to perform a specimen collection (nasal swab) if they became sick with symptoms of a cold or the flu. Participants also reported symptoms through weekly surveys. Symptoms included those common to acute respiratory infections and seasonal cold and flu-like illnesses (fever, cough, sore throat, shortness of breath, chills, fatigue, body aches, headache, nausea, and diarrhea). If a participant reported any symptoms on their weekly survey, they were immediately sent an email reminder to submit specimens. Specimens were tested for the presence of a panel of acute respiratory infections. Demographic information (age, gender, ethnicity, and location) and Twitter handle (optional) were also collected from each participant. Additional details of the protocol can be found in studies by Goff et al and Ray and Chunara [22,23].

We used the Tweepy application program interface (API) [24] to collect available tweets from participants, limited by the Twitter API, which only allows 3200 most recent tweets per user. The Twitter API only allows for collection of profiles that are shared publicly. These timelines were collected in March 2018. In addition, we collected data from a random set of Twitter users for comparison. These timelines were obtained in October 2018. We identified all users in a 2-week, 1% random Twitter stream and randomly selected users from this sample. Users were kept in the final random dataset if we were able to obtain tweets back to the start of the GoViral study (n=118). This was done to allow for matching between study participants and the random sample. However, this decision does bias the dataset away from very prolific users.

### Keyword Analysis and Topic Modeling

To answer RQ1, we identified tweets that explicitly referenced the individual’s current health status, focusing on colds or flu-like illness. As the number of individual tweets precluded manual coding, we used a keyword filtering approach. This is a common approach to increase the fraction of relevant instances [25-28]. We queried all timelines for tweets that included the following keywords:

- General words: flu, sick, throat, hurt, sinus, influenza, stomach, tummy, respiratory, nose, feeling, cold, feel, h1n1, h3n2, h5n1, flua, flub, infection, ill
- Symptoms: fever, cough, congested, stuffy, headache, ache, sore, head, phlegm, sneeze, asthma, pneumonia
- Medications: medicine, dayquil, nyquil, tamiflu, mucinex, theraflu, tylenol, motrin, aleve, naproxen, ibproufen, acetaminophen, advil, virus, oseltamivir, peramivir, infection, zanamivir, antiviral, guaifenesin, robitussin, phenylephrine, decongestant, pseudoephedrine, antihistamines

For use in the analyses described in the following section, we extracted topics from all tweets using latent Dirichlet allocation (LDA) [29] and a Gibbs sampling implementation with automatic hyperparameter optimization described in the study by Paul and Dredze [30]. Before feature extraction, all tweets were preprocessed in the same manner: usernames and URLs were replaced with generic tokens and emojis, nonalphanumeric characters, and extra letters were removed (eg, *greaaaat* is truncated to *great*). The Gibbs sampler was run for an initial 1000 iterations, and 100 samples were collected at the end and averaged to estimate the model parameters. The number of topics was set to 100. Each of the 100 topics has a distribution over words, characterizing the content of the topic, and each tweet has a distribution over the 100 topics. The topic probabilities in each tweet are used in the predictive models to describe tweet content.

### Predictive Modeling

To answer RQ2, we created several training and testing datasets (n=100) because the overall GoViral dataset was small. For each, 90% (73/81) of the eligible GoViral participants were randomly selected to be in the training set. The remaining 10% (8/81)were reserved for the test set. Using this method instead of creating one training/testing dataset allowed us to measure the robustness of the models on a number of datasets and generate summary statistics (area under the curve [AUC] and *P* values reported below). We then constructed 3 datasets.

The first dataset was used to discern if we could identify when participants were sick. For each participant, we randomly sampled one survey to include in the dataset. If that survey had no symptoms, we then randomly sampled another survey from the selected participant that had at least one symptom. Conversely, if the survey did report a symptom(s), we randomly sampled a survey with no symptoms. In this fashion, we balanced the number of asymptomatic and symptomatic data points and balanced the number of surveys per participant (to avoid bias from individuals who were particularly prolific survey respondents).

The second and third datasets were used to measure differences between the GoViral dataset and the Twitter random sample (RQ3). Here, each survey selected initially was matched with two additional data points. For each survey, we selected a random date during which the user tweeted but did not return a survey within a week on either side. We also selected a random date from the Twitter users collected at random. These datasets allowed us to measure if a GoViral user would return a survey in a particular week and if an individual was in the GoViral dataset. The purpose of this dataset was to measure if there was evidence of external factors that impacted study participants; for example, it could be that individuals were more likely to return surveys with symptoms because they stayed home when ill and had more time to fill out the survey.

For all predictive models, two types of features were used: topic features and behavior features. To construct topic features, we obtained all tweets for 1 week before the date of interest (eg, the date a survey was returned). We then obtained the topic distribution for those tweets and used the average of the topic distributions as a 100-dimensional feature vector. We selected a week (as opposed to other time frames) because the incubation of common flu and cold illnesses is approximately 1 to 4 days [31]. As such, 1 week is an appropriate buffer around the date of interest. We used the average topic distribution instead of individual tweet distributions because this allowed us to have the same dimensional feature vector for each user.

Behavior features included the (1) number of *@* mentions, (2) number of retweets, and (3) daily tweet frequency. All features were averaged over the previous week. These metrics have been used in prior research to describe information dispersal [32], information communication between friends [33], and user behaviors such as response rate for question-answering behaviors on social media [34]. When using features to distinguish between the GoViral sample and the random Twitter sample, we used the raw values. When using the features to identify user differences within the GoViral sample, values were Z-score normalized by the user (µ=0, ρ=1).

We built regression models to predict the symptoms of an individual using the topic and behavior features derived from Twitter. We used a binary logistic regression classifier built in Python 3.6.3 (Python Software Foundation) to predict whether or not a report contains at least one symptom using the implementation from Scikit-learn (version 0.19.1) [35]. Tenfold cross-validation on the training data was used to select the regularization parameter (using a grid search of values between 0.000001 and 100,000 in orders of magnitude). We also built individual classifiers for each symptom reported. Here, we included a survey in the positive class if it included the symptom of interest, regardless of if it also included other symptoms.

In addition to binary prediction, we used linear regression to predict the number of symptoms reported (a proxy for the severity of illness). Ridge regression using the Scikit-learn implementation [35] was used to force coefficients to be small while keeping all features. The regularization parameter for ridge regression was selected using 10-fold cross-validation on the training data. This study was approved by the University of Colorado Boulder institutional review board (protocol number 17-0470).

## Results

### Cohort Description

Overall, 396 individuals participated in the GoViral project and shared their Twitter handles, of which 186 returned at least one survey. Study participants returned 6.4 surveys on average, resulting in a total of 1283 surveys. Of these 1283 surveys, 417 included a report of at least one symptom. Participants were geographically widespread, representing 43 different states in the United States. Most participants were from New York, California, Texas, Washington, Massachusetts, Florida, New Jersey, and Virginia.

Of the original sample of 396 individuals, Twitter data were unavailable for 84 because they had private accounts (n=25), had never tweeted (n=4), or because the Twitter handle provided did not exist on Twitter at the time we collected data (n=55). Moreover, of the remaining sample, only 81 could be included in the final dataset as we required that any included individual returned both a survey with no symptoms and a survey with at least one symptom.

Demographic information (gender and ethnicity frequencies and mean age) for the overall GoViral dataset (*original data*) and the final set of individuals included in this study (*study cohort*) are shown in Table 1. Two individuals in the study cohort did not respond to demographic questions. Individuals in the original study were allowed to select multiple ethnicities; therefore, total across all ethnicity categories is greater than the number of individuals. Demographic distributions between the original data and study cohort are similar, with notable differences. The study cohort had more women compared with the full GoViral sample; it had a higher proportion of individuals who identified as white and had a smaller proportion of individuals identifying as black. Among the study cohort, individuals tweeted an average of 613 times during the study for a total of 51,141 tweets. These individuals also returned a total of 343 surveys (4.2 surveys per person on average).

**Table 1:** Study demographics.

Variable		Study cohort (n=81)	Original data (N=396)
**Gender, n (%)**			
	Male	54 (30)	235 (38)
	Female	24 (67)	149 (59)
	Other	1 (1)	6 (2)
**Ethnicity, n (%)**			
	Black	1 (1)	18 (5)
	White	69 (85)	311 (79)
	Native	2 (3)	9 (2)
	Latino	2 (3)	32 (8)
	Islander	11 (14)	58 (15)
Age (years), mean (SD)		40.91 (14.01)	37.47 (14.24)

### Health Disclosure in Tweets

To answer RQ1, we examined the 436 tweets that included health-related keywords and were tweeted during the GoViral study period. Each tweet was hand-coded as relevant or not relevant; relevant means that the tweet appeared to be an authentic description of the individual feeling poorly, with no other explanation. Mentions of events outside of infectious disease that could account for feeling ill were excluded (eg, recent surgery, consumption of alcohol, and the temperature was cold). Each tweet was annotated by two of the authors, and disagreements were resolved by the remaining author. Cohen kappa values were 0.66, 0.60, and 1.0 between the three pairs of annotators.

This process resulted in only 26 health-related tweets that could potentially be attributed to seasonal cold or flu viruses. Of these, only 2 were tweeted within 2 weeks (1 week before or 1 week after) of a positive symptom survey.

Overall, we found that health tweets were a small percentage of the tweets written near a positive symptom survey (only 2 tweets, 0.0039% of all tweets in the dataset). In the overall dataset, users tweeted 35 times a week on average (95% CI 34.6-35.3). We found that even among people who were active on Twitter and reported feeling sick, it was rare for them to actually tweet about sickness.

### Symptom Prediction

Results of the binary models are presented in Table 2.

**Table 2:** Logistic regression model results.

Outcome of interest and feature set				Area under the curve		*P* value
**Was an individual ill?**						
	**Any symptom**					
		Topic model	0.51		.38	
		Behavior features	0.30		<.001	
	**Body aches**					
		Topic model	0.57		<.001	
		Behavior features	0.50		—^a^	
	**Runny nose**					
		Topic model	0.47		.02	
		Behavior features	0.50		—	
	**Leg pain**					
		Topic model	0.47		<.001	
		Behavior features	0.50		—	
	**Nausea**					
		Topic model	0.52		.11	
		Behavior features	0.50		—	
	**Vomiting**					
		Topic model	0.50		—	
		Behavior features	0.50		—	
	**Sore throat**					
		Topic model	0.46		<.001	
		Behavior features	0.50		—	
	**Shortness of breath**					
		Topic model	0.50		—	
		Behavior features	0.50		—	
	**Fever**					
		Topic model	0.51		.28	
		Behavior features	0.50		—	
	**Fatigue**					
		Topic model	0.50		—	
		Behavior features	0.50		—	
	**Diarrhea**					
		Topic model	0.48		.27	
		Behavior features	0.50		—	
	**Cough**					
		Topic model	0.47		<.001	
		Behavior features	0.50		—	
	**Chills**					
		Topic model	0.48		.002	
		Behavior features	0.50		—	
**Was an individual a GoViral participant?**						
	**GoViral participant**					
		Topic model	0.67		<.001	
		Behavior features	0.50		—	
**Did the participant return a survey in the week of interest?**						
	**Returned a survey**					
		Topic model	0.50		—	
		Behavior features	0.50		—	

^a^Instances where *P* value cannot be calculated.

Table 2 shows the average AUC for all 100 models built, along with the *P* value for each (calculated using a *t* test, with a null hypothesis of H_0_=0.5). The AUC is a measurement of how well the model is able to correctly classify the outcome. An AUC of 1 would be a perfect classifier, whereas an AUC of 0.5 is a classifier operating at chance. An AUC less than 0.5 is a classifier operating worse than chance. It was not possible to predict if a user would return a survey with at least one symptom with logistic regression using the topic features (AUC=0.51; *P*=.38); however, it was significantly predictable using user behavior features, with a small effect size (AUC=0.53; *P*≤.001). There were only a few instances where individual symptoms were predictable using our models, and none when using the behavior features. When using topic modeling features, body aches were significant (AUC=0.57; *P*≤.001), and nausea and fever were nonsignificant but had AUC values over 0.5 (AUC=0.52; *P*=.11 and AUC=0.51; *P*=.28, respectively).

No relationship existed between either feature set and the number of symptoms using a ridge regression analysis (tweet topics: *r*=−9.03; Twitter behaviors: *r*=−0.05). Typically, negative *r* values indicate the model was overfit. However, in this instance, the models always selected the most aggressive regularization parameter, meaning all coefficients were extremely close to 0. Thus, we interpreted this finding to show that the number of symptoms reported (a proxy for illness severity) was not predictable using either feature set.

### Cohort Bias

To answer RQ3, we considered how this study cohort might differ from a sample selected at random from Twitter (see Table 2). When using topic model features, the random sample and the GoViral sample were predictably different on held-out data (AUC=0.67; *P*≤.001). Table 3 shows the most common topics associated with those in the GoViral sample compared with the random Twitter sample. Topics appear in the table if they were associated with at least one-third of the models built. The last column denotes which cohort the topic was associated with. In terms of themes, all the topics associated with science, research, or health were associated with the GoViral sample.

Importantly, the two samples were indistinguishable using behavior features (AUC=0.50; *P*=.19). In addition, it was not possible to predict if a GoViral participant returned a survey in a given week (AUC=0.5 with both feature sets). Thus, we found that there were no observable differences in tweet content or Twitter use patterns in weeks that participants returned a survey compared with the weeks they did not.

**Table 3:** Most important topics for the in-sample classifier and direction of association.

Topic	Top words	Associated with
13	science new human scientists data microbiome learning research study using great lab dna brain gt machine biology paper talk work wcsj2017 project interesting cool ai deep citizen check bacteria	In sample
24	cancer study disease research new risk brain join heart treat-ment scientific patients contributed health pain blood humanitar-ian drug help gut therapy depression diseases flu high dr women years vaccine	In sample
36	spread help share awareness terrible disease time cpu wcg earned points donating results days word donated past wcgrid week month years day hours son old semicolon badge 3026 1650935 raise	In sample
97	5points genes gene human dna cancer notes new data cells genome tumor cell variants genomes vs bog15 genetic rare finds expression nygc rna non agbt15 gt pg14 protein paper	In sample
16	gold olympics usa olympic ich medal die org und silver team der ist rio2016 old medals contact es ein hockey won win das teamusa women nicht wins war einen	Random sample
29	que la el en se es lo por los mi para una te del las ya si como pero todo ser yo su tu da eu os est qu hoy	Random sample
38	hai a1 ho ke india ki modi a3 ka a2 se hi a5 nahi kya ko bhi toh a4 na timepass aur ab main contest mein tu ye kar	Random sample
52	new photo facebook posted martin instagram king photos luther video page yorker album pic shoot jeff caption cover credit selfie beijing york shkreli beatbaker burger ad repost fb likes selfies	Random sample
53	follow retweet gain trapadrive followers fast let thanks appreci-ate gainwithxtiandela retweets 1ddrive likes tweet active time rts naijafollowtrain follows 500 bam gainwithpyewaw ifb gaining turn 100 quick mzanzifollotrain gainwithtrevor	Random sample
69	launch shared rocket sd first spacex holbrook falcon test elon musk space satellite ship says fund 10 percent barrier mission join landing location second stage project life new cruise	Random sample

## Discussion

### Principal Findings

This study found that there were instances of self-disclosure of flu-like symptoms on social media that correspond to disclosed survey symptoms, but they were exceedingly rare. Although it has always been obvious that only a fraction of people disclosed their health status on the internet, that fraction has not previously been quantified for flu-like illness. Out of 426 self-reports of illness, only 2 coincided with a user tweeting about their own poor health.

The fact that self-disclosure of flu-like illness on Twitter happens so rarely, even among active Twitter users, opens the possibility that there is a selection bias in terms of who chooses to disclose this information on the internet when they are feeling ill. Whether such a bias exists, as well as its characteristics, has not been measured to date. Unfortunately, this study was not able to provide more insight into this potential effect because of the very small number of disclosures in our dataset. Importantly, prior work has not observed this bias, and future work attempting to better characterize this will need to recruit a large number of participants to effectively measure it.

In addition to identifying disease mentions, we attempted to predict disease state from users’ tweet content (using topic models) and social media behaviors. Our models were not able to predict if an individual would return a symptomatic survey from their tweet content alone. Our study found that behavior features (the frequency of tweets, retweets, and @ mentions) were significantly but only slightly predictive of illness, and this effect was only present with classification, not regression.

This is in contrast to work that found that social media post content might be related to illness status. Smith et al recruited participants from an emergency department and correlated health conditions with posting frequency on Facebook [20]. Topics on Facebook, ascertained through LDA were also examined in relation to posting frequency. Although the actual correlation coefficients were small, they found that individuals who posted more often tended to have more complaints such as *headache* and *sick* in comparison with the infrequent posters who used words such as *birthday* and *enjoy* [20].

Finally, efforts to individually validate infectious disease mentions on the internet are further complicated by multiple additional sources of bias. The 81 individuals included in this study were biased from the original GoViral dataset (Table 1). In addition, although the GoViral cohort certainly included active Twitter users (tweeting an average of 35 times per week), the respondents were not representative of all Twitter users, in particular with respect to their tweets’ topics. We found that the study participants discuss topics about science and health more frequently, whereas more diverse topics (eg, those about sports and social media) were more predictive of the random sample. This could indicate that those in the GoViral population were more interested in public health problems than the average Twitter user. However, we found the 2 populations to be indistinguishable based on their Twitter use behaviors. Those in the random sample and the GoViral samples used Twitter with similar overall frequencies and with similar hashtag and @ mention frequencies.

It is well known that internet data are demographically biased [36-38], for example, social media platforms are typically biased toward young adults compared with the elderly [37]. Prior work has also demonstrated that subsets of Twitter data are also biased; for example, Sloan et al showed that geotagged tweets are not representative of the Twitter base [38]. Taken together, this illustrates the numerous levels of bias that those who work with social media data face.

Recruitment bias is known to happen in most cohort-based studies and has been shown in a variety of contexts, including twin studies [39], physical activity studies [40], and paid vs unpaid studies [41]. More recent research has shown that studies recruiting using online data also experience this bias [42,43]. However, this is the first study, to our knowledge, to explore recruitment bias on a social media platform for infectious disease research.

### Limitations of the Study

As noted above, the sample size of this study is a substantial limitation. We found only 2 instances of tweeting about illness while sick from a collection of 396 participants who shared their Twitter handles. However, it should be noted that it would be labor- and cost-intensive to amass ground truth data at a much larger level, and it may be especially difficult to collect enough data in this domain. To obtain a sizeable number of instances where users tweet about illness they are sick, one may have to scale up recruitment efforts [22] or define more specific inclusion criteria. In addition, we noted that we observed some trends in social media behavior and disease severity that would be worth testing in a larger sample with greater statistical power. We discuss the trends we observed in exploratory analyses in Multimedia Appendices 1 and 2.

As it was not required that individuals return a survey each week of the study, it is impossible to ascertain if there are response biases associated with survey response. We attempted to measure this by building the classifier to predict if a user responded in a given week. This outcome was completely unpredictable by our feature sets, but it is still possible that there were unmeasured differences.

We also noted the substantial number of individuals in the original GoViral dataset who could not be included because we were unable to obtain their Twitter data. It is possible that individuals with private accounts disclose illness at different rates than those with public accounts, and it is impossible to measure that with this dataset.

Finally, we acknowledge the possibility that our keyword-based procedure for identifying health-related tweets may have missed relevant tweets, which thus would have been excluded from our analysis. We attempted to reduce this risk by using a large set of terms, including very general words such as *feel* and *feeling.*

### Conclusions

Overall, we did not find strong evidence that health status with respect to cold and flu-like illness can be predicted from tweet content or behavior. A larger and more representative study would help verify this on a broader scale. However, in general, we posit that verifiable traces of illness on the internet might be rarer than initially believed by the social media monitoring community. It is possible that there may be an informative signal from social media platform behaviors (eg, tweet frequency) for individual health status that would be interesting to study in a larger dataset. Finally, we demonstrate a clear recruitment bias that should be considered when building large ground truth datasets for the infectious disease domain.

## Appendix

Multimedia Appendix 1 Normalized tweet frequency near a survey. Normalized tweet frequencies (y-axis) are shown with respect to the number of days before or after a survey (x-axis), where day 0 is the day a survey is returned on. Data are stratified by the number of symptoms. Lines show the average value and shaded regions represent the 95% confidence interval. This figure was generated by comparing the normalized tweet frequency of users in the week prior to and the week after a survey response. We stratify by the number of symptoms reported by a user in order to observe the effect of illness severity on tweet frequency. In some cases (e.g., Figure S1 at ≥ 5 symptoms), the differences are nearly statistically significant, though they are never actually significant on the day of a returned survey.

Multimedia Appendix 2 Normalized tweet frequency near a survey. Normalize tweet frequencies (y-axis) are shown with respect to the number of days before or after a survey (x-axis), where day 0 is the day a survey is returned on. Data are stratified by the symptoms reported. Lines show the average value and shaded regions represent the 95% confidence interval. This figure was generated by comparing the normalized tweet frequency of users in the week prior to and the week after a survey response. Data are stratified by the symptom reported by a user (where the survey is included if the symptom of interest is reported, regardless of if there are additional symptoms reported). There are some symptoms that show significant patterns, in particular leg pain, nausea, shortness of breath and chills or night sweats have sections that are statistically significantly different from users with no symptoms reported.

## Abbreviations

API: application program interface
AUC: area under the curve
LDA: latent Dirichlet allocation
RQ: research question
