# Supplementary figures and images for: Comparison of Social Media, Syndromic Surveillance, and Microbiologic Acute Respiratory Infection Data: Observational Study
Source: JMIR Public Health Surveill. 2020 Apr 24;6(2):e14986. doi: 10.2196/14986 (PMC7210500; doi:10.2196/14986)

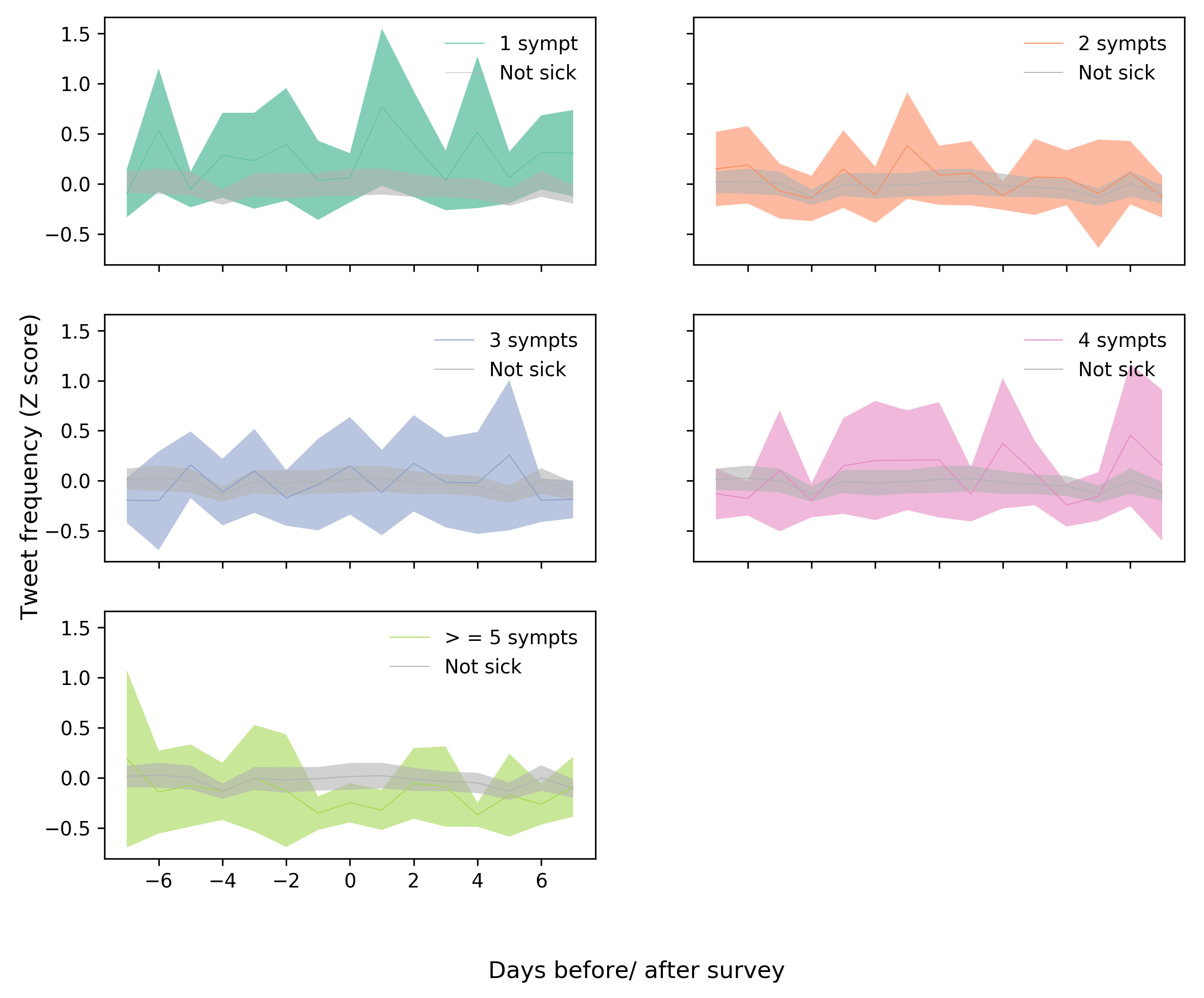

Supplement: Multimedia Appendix 1 [file publichealth_v6i2e14986_app1.png]

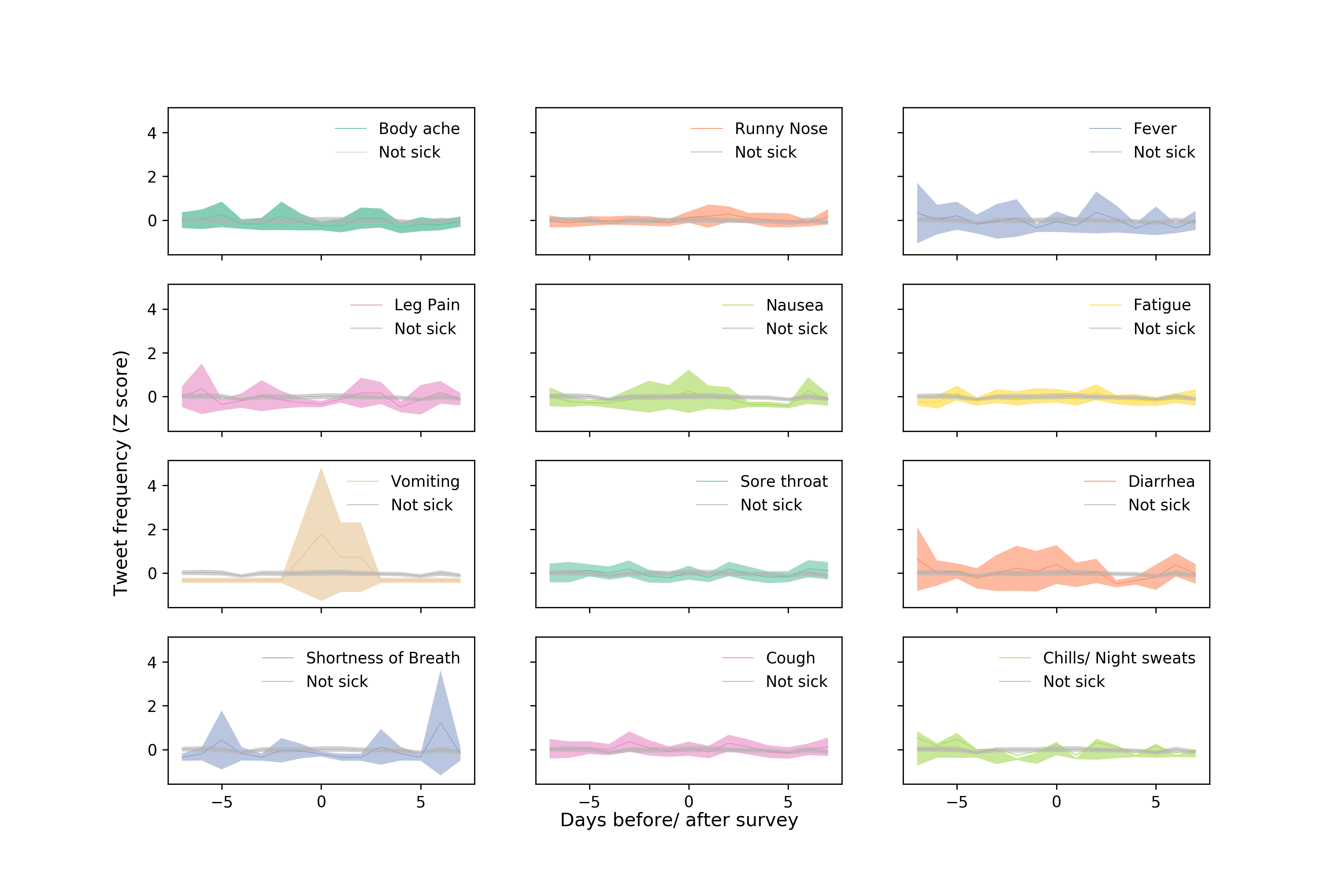

Supplement: Multimedia Appendix 2 [file publichealth_v6i2e14986_app2.png]
